# Progress in Medicine

**Published:** 1900-01-27

**Authors:** 


### Progress in Medicine.

### FEVERS.

**Typhoid.**—Among the recent epidemics the one at Lyons was ascribed by Gabriel Roux[1] to contamination of the water supplies, though the low level of the ground water at the time was particularly noticeable. The town supplies were affected by the introduction of a new set of filters, which were so imperfect that the colon bacillus was found to have passed through them, while in the well water the typhoid bacillus itself was discovered. Nägeli,[2] in an epidemic at Berne, utilised the condition of the blood as a means of diagnosis in addition to the serum test, and claims to be able to fix the time of a past attack of typhoid. He lays down that the neutrophile leucocytes are diminished during the first stage of the fever, and only regain the normal several weeks afterwards. The lymphocytes increase during the third stage and afterwards, and do not fall to the normal till after six weeks of apyrexia. The eosinophile cells vanish during the second stage, and do not return to the usual number till after three weeks or more of apyrexia. Complications such as otitis lead to an increase of neutrophiles, but have little effect on the other two varieties. Even where the Widal reaction fails the examination of the blood may give a certain diagnosis. We can summarise his rules as follows: The neutrophiles and eosinophiles decrease at an early stage and remain so for several weeks after recovery, while the lymphocytes, after an initial fall, rise above normal in the third stage and remain so for a similar period. Herman Biggs[3] finds the Diazo reaction valuable in the first days of fever before the Widal test is available, and as to vaccination mentions the report of the Maidstone Asylum, where none were attacked out of 95 vaccinated, while in 100 untreated there were 19 cases. W. H. Park notices the infectiousness of urine and fæces for several weeks after convalescence. This is, however, quickly put an end to by urotropine in doses of 10 grains three times a day. The urine is, however, free from bacilli till the third week of the fever. Janeway reports three cases of tetany during typhoid, but both this and herpes labialis are extremely rare complications. W. H. Thompson, in the same discussion, gave the typhoid results at the Roosevelt Hospital for the last year, during which 368 patients were treated, with a mortality of 6·8 per cent. Fifteen per cent. suffered from relapses. The cool bath was used as soon as the temperature reached 103 deg., and saline rectal injections were also given. The artificial Nauheim water was found best for the baths. A purge of calomel and jalap was given every third day during the first fortnight. The food consisted solely of milk and lime-water with pepsin. When the tip of the tongue became dry ♏x-xxx of spirits of turpentine were given every three hours. Fitz showed that the mortality at the Massachusetts General Hospital varied very little from 1849 to 1899, being from 13 to 16 per cent., and he finds that perforation, hæmorrhage, and relapse are as frequent now as formerly. In the Bellevue Hospital the last 104 cases showed only 9 6 per cent. of deaths. No solid food is there given until after eight or nine days of apyrexia. W. W. Keen notices that 150 cases of operation for perforation are now on record, and the recoveries of late have reached over 26 per cent. The best time for operation appears to be after the primary shock is over, generally in the second twelve hours after perforation. Cocaine anæsthesia has been preferred of late to chloroform or any other. In London there has been a great increase of typhoid, which reached twice the normal rate in November,[4] but whether this was owing to climatic causes, to shellfish or the general water supply is doubtful. At Shoeburyness 30 cases occurred, which seemed due to cockles gathered from water infected with sewerage, the drainage, milk, and water supplies being uncontaminated. W. C. Bosanquet[5] reports 215 cases of typhoid treated in Charing Cross from 1889 to 1898, with a mortality of 18·5 per cent., the cause of the largest number of deaths being perforation. There were six ambulatory cases, all of which proved fatal. Cold sponging was used for high temperatures. In American cities the death-rate from typhoid is very high. Thus Crurn[6] shows that the rate of 24 cities during the years 1889-1898 averaged 41 per 100,000, whereas London and Berlin only gave rates of 13 and 7 per 100,000. However, in the last few years great efforts have been made to improve the water supply, with the result that the rates have been reduced very greatly. Thus Chicago shows a fall of 61 per cent.; but, nevertheless, nearly 42,000 persons perished in the 24 towns during the decade from this preventable disease. In the great outbreak which affected the troops during the Spanish war it was found necessary to move a hospital, and 104 patients were conveyed four miles to a new site without a single death, and marked subsequent improvement.[7] This fact is of some value in connection with the risk of removing patients from their homes to a fever hospital. Dublin, again, shows a marked outbreak, the *Lancet*[8] mentioning more than 1,100 cases, of which, perhaps, 100 were due to one infected supply of milk.

[1] Med. Chron., June. [2] Med. Chron., Nov. [3] Med. Rec., Oct. 28. [4] Lancet, Nov. 25. [5] Brit. Med. Jour., July 8. [6] Med. Rec., Aug. 12. [7] New York Med. Jour., Aug. 5. [8] Lancet, Nov. 25.

### DISEASES OF THE THYROID.

**Sporadic Cretinism.**—Bramwell[1] records a well-marked case of myxœdema, in which the patient menstruated regularly. The patient was 36 years of age, and measured exactly 36 inches in height. Her appearance was typical of a marked degree of sporadic cretinism. Menstruation began at the age of 25 years, and had continued every month. The nipples and areolæ were somewhat developed, and there was a fulness in the position of the breasts. There were no pubic hairs. Her intellectual development was that of a child of five or six years. The existence of menstruation in well-marked sporadic cretinism is quite exceptional, for in the great majority no development of the sexual organs takes place.

**Myxœdema.** — Chapman[2] describes three cases of myxœdema, and makes some remarks on the diagnosis in the early stages of the disease. Obstinate anæmia, even in a young woman, may be a forerunner of myxœdema, and the supervention of any mental dulness should give rise to the suspicion of myxœdema. The existence of a certain "sloppiness" of the conjunctiva, which is best observed by pushing up the lower eyelid at the outer corner of the eye, is a valuable sign, for it generally denotes either Bright's disease or myxœdema, and if the urine is normal the latter disease is probably present.

Buchanan[3] has recently treated a case of myxœdema with "colloid material," which, according to the investigations of Hutchison, contains the active substance of the thyroid gland. The material used was a "palatinoid" preparation, each dose being equivalent to five grains of the fresh gland. The results were excellent, and the patient remains now in health by taking one or two doses daily.

**Etiology of Exophthalmic Goitre.**—Carter[4] discusses the etiology of exophthalmic goître in an interesting paper. He says that against the sympathetic nerve lesion hypothesis it may be urged that the pathological alterations found are not peculiar to the disease, and that only some of its symptoms can be so explained. An over-activity of the thyroid gland is often assumed to be the cause of exophthalmic goître, but though this theory may account for many of the observed symptoms exophthalmos has never been produced by the administration of thyroid gland.

It is, therefore, suggested that the disease is possibly an acute infection by some micro-organism. In favour of this the following arguments may be put forward: Simple goître has been hitherto thought to be due to excess of calcic, or magnesic, or other salts in the drinking water, but recently Grasset has found special parasitic elements in the blood of eight patients suffering from recent goître. These micro-organisms were spherical bodies, larger than blood corpuscles, having no nucleus, and containing red pigment and possessing a free flagellum.

Then, again, cases are occasionally reported in which exophthalmic goître runs a very acute course. Thus Lloyd reports one in a patient, previously quite healthy, who died in three days with all the symptoms of the disease. Such cases suggest strongly an acute poisoning. There is also a certain predominance of the disease in certain localities, *e.g.*, Kent, Surrey, Wiltshire, and the Thames Valley.

**Treatment of Exophthalmic Goitre.**—Pitres[5] has recently published the results obtained by treating exophthalmic goître with injections of an iodoform solution in ether. The dose is one cubic centimetre injected into the goître at intervals of about eight days. The injections must not be made more frequently on account of the swelling provoked in the gland, which lasts for some days. The pain caused is acute, but it lasts for about 12 minutes only. In a very short time after the first injection the nervous symptoms cease, sleep returns, the gland hardens little by little and becomes harder, and the exophthalmos disappears. The patient continues to suffer for some time from cardiac irritability, but the arterial pulsations diminish, and the sensations of palpitation disappear. Twelve patients have been treated, but only six of these have been followed up enough to draw conclusions of value. These six have remained well for over two years, and none of the injections carried out upon them was followed by any untoward result.

**Thyroid Treatment of Obesity.**—Ebstein[6] is of opinion that the thyroid treatment of obesity is unsatisfactory in its practical results, because the loss of weight is inconstant, often fails, and in those cases in which it does take place recurs when the treatment is omitted or during its long-continued use. Loss of weight so obtained is not accompanied by that advance in general health which is observed in cures based on diet and appropriate rules of living. Furthermore, the loss of weight obtained through thyroid preparations is not rational, since the body loses albumen as well as fat. He concludes that thyroid extract is not needed in the treatment of obesity, since good results can be obtained by other methods and without accompanying dangers.

**Operative Treatment of Goitre.**—Kocher,[7] of Berne, has published the results of 600 cases in which thyroidectomy has been done. The operation is an absolutely safe one in uncomplicated cases of simple goître, and is held to be indicated by nodular growths in the thyroid gland in all cases of cystic disease, and whenever there is a suspicion of malignancy. One of the chief indications for operation is dyspnœa due to compression of the trachea, for this cannot be remedied by medicinal treatment. As the only danger of thyroidectomy in simple cases of goître is the fatal action of a general anæsthetic, cocaine injected locally is always used except in young children and very sensitive subjects. A curved skin incision with its convexity downwards is made across the front of the neck. The sternohyoid and sternothyroid are detached at their lower extremities from the sternum. The enlarged gland, after division of its fibrous capsule, is raised from the trachea and drawn out of the wound, so that the thyroid vessels are put on the stretch before being ligatured. The isthmus, after its exposure and after ligature of its vessels, is compressed by forceps, and reduced to a narrow band. Some portion of the gland is almost invariably left to prevent the occurrence of myxœdema. The operation was fatal in 6 out of the 18 cases of malignant disease, in 2 of the 11 cases of strumitis, and in 2 of the 15 cases of exophthalmic goître. Of the remaining 556 cases operated on for simple goître one only was fatal, and this from the effects of chloroform. The high death-rate of the operations for malignant disease of the thyroid is due to the fact that portions of important structures—*e.g.*, the carotid artery, pneumogastric had to be removed. Cases of malignant growths in the thyroid have been reported by Firth[8] and by Barker,[9] the former being a sarcoma and the latter a papillomatous carcinoma. Cases of cysts of the thyroid have been described by Heath[10] and by Syms.[11]

**Parathyroid Glands.**—Welsh[12] finds that removal of the four parathyroid glands in the cat leads to acute and severe symptoms, with a rapidly fatal issue, even though the thyroid be retained practically uninjured. Removal of three parathyroids does not lead to death, but may cause transient symptoms similar to those which result from removal of the two glands; loss of two parathyroids does not produce any appreciable change. Removal of the thyroid and some of the parathyroids may lead to death with acute symptoms if only one parathyroid is left, but may not induce any obvious derangement if two parathyroids are retained —at least, not for several months. Mouth administration of the fresh parathyroid of the ox has no effect in mitigating the symptoms or in averting death after removal of the thyroid and parathyroid in the cat, even though relatively enormous doses are given.

[1] Bramwell, Lancet, Dec. 12, 1898. [2] Chapman, Lancet, Sep. 30, 1899. [3] Buchanan, Brit. Med. Jour., June 17, 1899. [4] Carter, Edin. Med. Jour., Oct., 1899. [5] Pitres, Lancet, Aug. 19, 1899. [6] Ebstein, Deut. Med. Woch., 1899, Nos. 1 and 2. [7] Kocher, Med. Press, Dec. 14, 1898. [8] Frith, Lancet, Aug. 28, 1899. [9] Barker, Brit. Med. Jour., Jan. 21, 1899. [10] Heath, Pract., Sep., 1899. [11] Syms, Med. Rec., April 2, 1899. [12] Welsh, Jour. of Path. and Bact., vol. x.

